# Interruption in physical activity behavior among Chinese adults in 2015 and 2020: trends and multilevel correlates based on two national surveys

**DOI:** 10.1186/s12889-026-26760-3

**Published:** 2026-02-25

**Authors:** Yibo Gao, Mingzhe Li, Koya Suzuki, Yanfeng Zhang, Yichuan Tian, Xiang Pan, Lupei Jiang, Donghai Xu, Qiaoyu Yang

**Affiliations:** 1 China Institute of Sport Science, Beijing, China; 2 Graduate School of Health and Sports Science, Juntendo University, Inzai, Japan; 3 Institute of Health and Sports Science & Medicine, Juntendo University, Inzai, Japan; 4 Juntendo Administration for Sports, Health and Medical Sciences, Tokyo, Japan; 5 School of Politics & Public Administration, Soochow University, Suzhou, China

**Keywords:** Physical activity, Behavioral interruption, Adults, Influencing factors, National survey

## Abstract

**Background:**

While physical activity (PA) promotion is a global priority, behavioral interruption remains a significant challenge for long-term maintenance. This study aims to analyze the trends and multilevel correlates of interruption in PA behavior among Chinese adults using national survey data from 2015 to 2020, providing a basis for targeted public health interventions.

**Methods:**

Data were drawn from the 2015 and 2020 National Fitness Surveys that included 31 provinces (autonomous regions and municipalities) in China, including 120,067 adults aged 20–59 years (54,854 in 2015 and 65,213 in 2020). χ² tests were used to compare the rates of interruption in PA behavior. Generalized linear models were used to examine the individual-, family-, and community-level factors associated with interruption in PA behavior. A pooled analysis with interaction terms (survey year × correlates) was performed to formally test the changes in association strength between 2015 and 2020. The covariates included age, sex, residence, education, occupation, and marital status.

**Results:**

The prevalence of interruption in PA behavior was higher in 2020 than in 2015 (16.4% vs. 6.8%, χ²=2596.723, *p* < 0.001). At the individual level, lack of interest in PA (2015 odds ratio [OR] = 1.64, 95% confidence interval [CI]: 1.37–1.93; 2020 OR = 1.51, 95% CI: 1.32–1.73), feeling lazy about PA engagement (2015 OR = 1.16, 95% CI: 1.03–1.29; 2020 OR = 1.17, 95% CI: 1.05–1.31), and perceiving a good physical condition (2015 OR = 1.24, 95% CI: 1.04–1.49; 2020 OR = 1.11, 95% CI: 1.00–1.24) were positively associated with interruption in PA behavior. Receiving professional guidance was negatively associated with interruption (2015 OR = 0.87, 95% CI: 0.79–0.96; 2020 OR = 0.73, 95% CI: 0.55–0.93). At the family level, household responsibility in 2020 (OR = 1.13, 95% CI: 1.01–1.34) and work demands (2015 OR = 1.08, 95% CI: 1.02–1.15; 2020 OR = 1.19, 95% CI: 1.05–1.42) were associated with a higher likelihood of interruption in PA behavior. Childcare responsibility showed a modest positive association in 2020 (OR = 1.11, 95% CI: 1.02–1.22). At the community level, perceived lack of convenient access was negatively associated with interruption in PA behavior (2015 OR = 0.80, 95% CI: 0.64–0.95; 2020 OR = 0.85, 95% CI: 0.79–0.92). Lack of suitable facilities in 2020 (OR = 1.22, 95% CI: 1.10–1.33), lack of organized groups or teams in 2020 (OR = 1.33, 95% CI: 1.21–1.47), and financial constraints in 2015 (OR = 1.09, 95% CI: 1.03–1.16, *p* = 0.047) were positively associated with interruption. Interaction analyses confirmed that the associations for professional guidance, lack of interest, and household responsibilities significantly changed between 2015 and 2020 (all *p* < 0.05).

**Conclusions:**

Between 2015 and 2020, interruption in PA behavior among Chinese adults increased substantially. Multiple-level factors (individual, family, and community) were associated with the interruption patterns. Then, structural shifts in correlates over time. Understanding these characteristics may help inform the development of public health strategies that support sustained PA engagement.

## Introduction

Regular and sustained participation in physical activity (PA) is associated with numerous health benefits [1], including lower risks of cardiovascular disease [2], type 2 diabetes [3], cancer [4], and premature mortality [5]. Although global initiatives continue to promote PA engagement, maintaining regular activity remains a significant public health challenge [6, 7]. However, many individuals discontinue PA after initially establishing exercise routines [8], reducing behavioral continuity, diminishing health gains, and even returning to a sedentary or inactive state. This pattern suggests that interruption in PA behavior is a distinct behavioral dimension rather than a simple absence of PA [9].

While sedentary behavior describes insufficient movement at a specific time point, interruption in PA behavior refers to the discontinuation of an already-established exercise habit. This phenomenon reflects a dynamic behavioral process linked to individual motivation and psychological states, social support, environmental characteristics, and broader policy contexts [10–12]. However, most existing studies have focused on PA initiation or promotion, with comparatively limited attention paid to the maintenance of or interruption in established exercise behaviors [13]. Moreover, research exploring the psychological, social, and environmental correlates of PA maintenance has typically relied on region-specific samples or short observation periods [14–16], making it difficult to characterize how structural and environmental changes relate to interruption in PA behavior over time.

To address the limited evidence on interruptions in established PA behavior, this study used data from the 2015 and 2020 National Fitness Surveys, which applied consistent sampling and identical questionnaire items, including the same definition of interruption in PA behavior. This methodological alignment ensures strong comparability between the survey waves. The period between 2015 and 2020 also represents a critical phase of national PA promotion in China, during which major policies such as the National Fitness Plan (2016–2020) [17] and Healthy China 2030 [18], substantially expanded public sports facilities and community exercise resources [19]. These structural improvements form an important policy context for interpreting the changes in adults’ PA behavior [20]. Additionally, the 2020 survey was conducted months after the initial COVID-19 outbreak. Although strict restrictions were lifted, earlier disruptions in daily routines, remote working arrangements, and temporary closures of public exercise venues may have influenced activity patterns [21]. Although causal inference is not possible, this context is relevant for understanding the 2020 data.

Understanding why individuals cease their established routines is critical for health, yet interruption in PA behavior remains under-researched compared to PA initiation. This study aims to examine the trends and multilevel correlates of interruption in PA behavior among Chinese adults using national survey data from 2015 to 2020. Specifically, we analyze how individual-, family-, and community-level factors influence the maintenance of exercise routines. These findings will inform targeted public health strategies to prevent behavioral relapse and promote sustained PA participation.

## Methods

### Data source

**Fig. 1:**
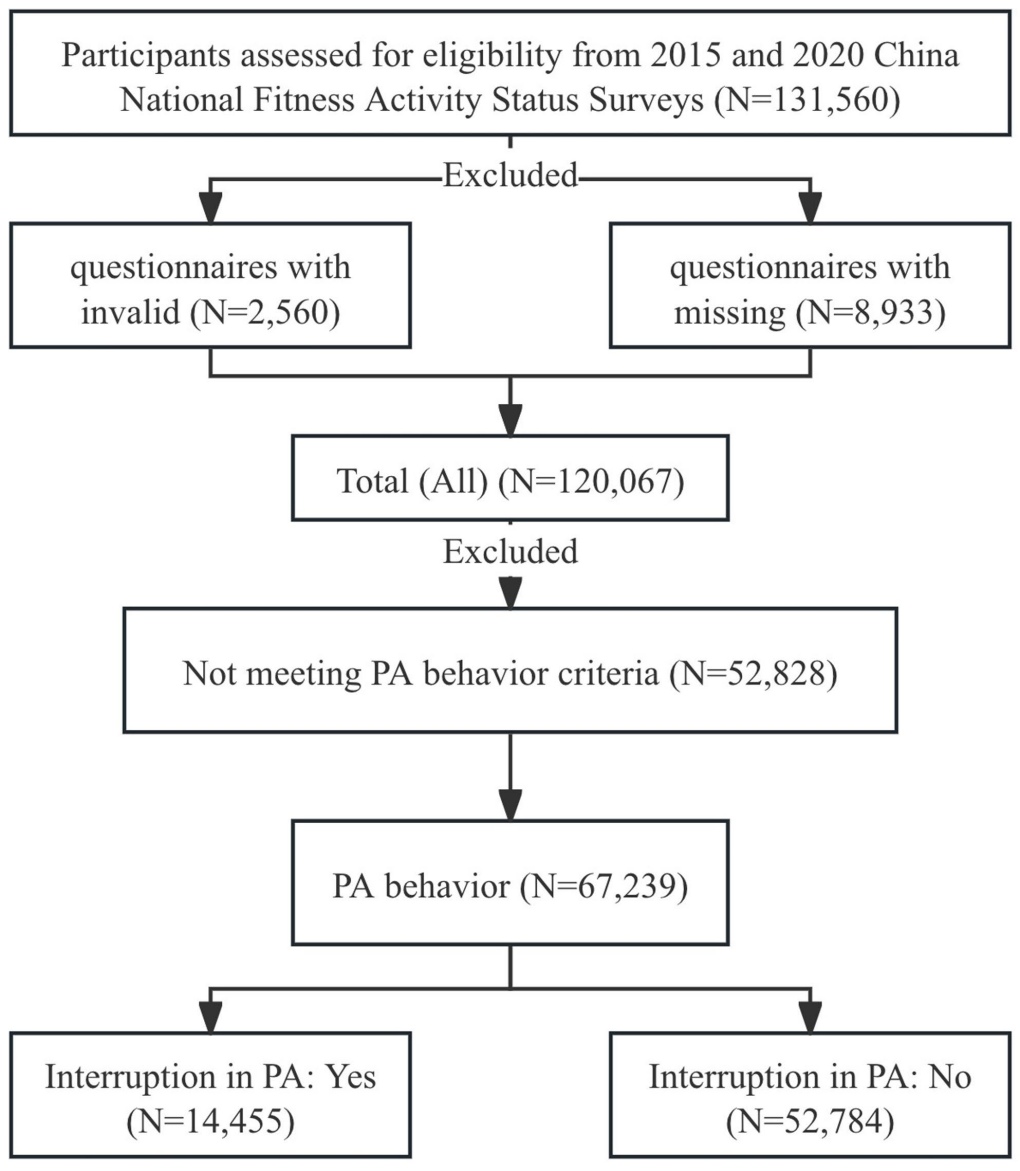
Flow diagram of participant selection and exclusion

Data for this study were obtained from the 2015 and 2020 waves of the China National Fitness Activity Status Survey, which included adults aged 20–59 years across 31 provinces, autonomous regions, and municipalities in China. The two surveys were conducted from January 1 to April 30, 2015 [22], and from September 1 to November 30, 2020, respectively [23]. A three-stage probability proportional to the size of the sampling method was employed. In the first stage, 10–20 county-level units were selected randomly from each provincial administrative unit. In the second stage, 13 village or neighborhood committees were randomly chosen from each selected county-level unit. In the third stage, four adults aged 20–59 years were randomly sampled from each selected village or neighborhood committee. Data were collected through household visits, and 11,493 questionnaires with invalid or missing information (8.7%) were excluded. A total of 120,067 participants were included, of whom 67,239 (56.0%) met the criteria for established PA behavior. Within this active population, 14,455 (21.5%) experienced interruption in PA. The flow of participant recruitment and selection is shown in Figure 1.

As shown in Table 1, Significant demographic differences were observed between the interruption in PA: Yes and the interruption in PA: No groups (all *p* < 0.001).

**Table 1:** Population distribution in 2015 and 2020 (n)

Groups	Stratification	Total		Interruption in PA		*p*
		All	PA behavior	Yes	No	
Total		120,067	67,239	14,455	52,784	
Age group (years)	20–29	27,857	16,642	3,682	12,960	< 0.001
	30–39	30,196	17,226	3,875	13,351	
	40–49	30,882	17,046	3,570	13,476	
	50–59	31,132	16,325	3,178	13,147	
Sex	Male	60,431	34,831	8,410	26,421	< 0.001
	Female	59,636	32,408	6,071	26,337	
Residences	Urban	68,539	42,728	9,230	33,498	< 0.001
	Rural	51,528	24,511	5,016	19,495	
Marital status	Unmarried	17,746	11,093	2,451	8,642	< 0.001
	Married	98,834	54,357	12,783	41,574	
	Divorced	2,404	1,313	291	1,022	
	Widowed	1,083	476	86	390	
Educational attainment	Primary school and below	19,049	7,607	1,663	5,944	< 0.001
	Junior high school	42,856	21,395	4,231	17,164	
	High school	27,933	16,860	3,281	13,579	
	Undergraduate	29,395	20,769	4,324	16,445	
	Postgraduate degree or above	834	608	135	473	
Occupation	Occupation 1	59,145	32,906	6,500	26,406	< 0.001
	Occupation 2	19,160	8,064	1,396	6,668	
	Occupation 3	26,087	15,424	3,169	12,255	
	Occupation 4	9,771	6,624	1,308	5,316	
	Occupation 5	5,904	4,221	946	3,275	

Note: Occupation 1: Temporary workers, unemployed, or unskilled laborers. Occupation 2: Manual workers and self-employed persons. Occupation 3: General management versus general professional and technical staff. Occupation 4: Middle management and middle-level professional and technical staff. Occupation 5: Senior managers and senior professional and technical staff. PA behavior refers to participants who met the exercise criteria (frequency ≥ 1 time/week, duration ≥ 10 min/session, and moderate-to-high intensity) as defined in the Methods. The Total (All) column presents the demographic profile of the entire study sample. The p represents statistical differences between the Interruption in PA: Yes and Interruption in PA: No groups, calculated using Chi-square tests

### Survey procedures

Before data collection began, training sessions were conducted for survey coordinators. These coordinators subsequently organized secondary training for field investigators to ensure consistency in understanding the survey objectives, procedures, and operational standards. During the survey, basic information about the sampling frame was obtained from local statistical departments and used for random sampling.

Data were collected through face-to-face household interviews. During the interviews, the investigators explained the purpose and significance of the survey, and written informed consent was obtained from each participant before the questionnaire was administered. Once the survey was completed, personal identifiers were processed using a three-level coding system to protect the confidentiality of each participant. The study protocol was conducted in accordance with the principles of the Declaration of Helsinki and was approved by the Ethics Committee of the China Institute of Sport Science (approval nos. CISSLA-20191029 and CISSLA-20220629).

### Variable selection

#### Dependent variable

PA behavior was assessed using three indicators from the China National Fitness Activity Status Survey Questionnaire developed by the General Administration of Sport of China: frequency, duration, and intensity per PA session during the past year. Frequency categories ranged from seven times per week to never; duration categories ranged from 10 min or less to 120 min or more; and exertion categories ranged from breathing and heart rate almost unchanged to markedly increased with noticeable sweating, with intensity reflecting subjective self-perceived exertion. PA behavior was classified as engaging in PA if they reported the frequency was at least once per week, the duration was at least 10 min per session, and the intensity was moderate or higher. This questionnaire has been used in multiple nationwide surveys since its first implementation in 1997 and has demonstrated high reliability and comparability [24].

Interruption in PA behavior was assessed using the following item: “In the past year, whether physical activity interruption (defined as having no physical activity behavior for at least six consecutive months) existed?” To ensure that this variable reflected the discontinuation of an existing PA habit rather than general inactivity, respondents who reported “never” for the PA frequency item were excluded from being classified as having an interruption. Thus, in this study, interruption specifically captured individuals who had previously engaged in PA but later stopped for at least six months.

#### Independent variables

The independent variables were derived from items covering individual-, family-, and community-level factors. At the individual level, seven aspects were included: receiving professional guidance, lack of interest in PA, feeling lazy about PA engagement, perceiving a good physical condition, worrying about being ridiculed, considering PA unnecessary, and concern about being injured. At the family level, three aspects were assessed: childcare responsibility, household responsibilities, and work demands. At the community level, four aspects were included: perceived lack of convenient access, lack of suitable facilities, lack of organized groups or teams, and financial constraints. Detailed questionnaire items and coding schemes are presented in Table 2.

**Table 2:** Sampling questions and options

Level	Code	Questions	Options
Individual level	Y1	Do you receive professional guidance related to physical activity?	0 = No, 1 = Yes
	Y2	Do you lack interest in physical activity?	0 = No, 1 = Yes
	Y3	Do you feel lazy about engaging in physical activity?	0 = No, 1 = Yes
	Y4	Do you believe your physical condition is good enough that you needn’t do physical activity?	0 = No, 1 = Yes
	Y5	Do you worry about being ridiculed by others for engaging in physical activity?	0 = No, 1 = Yes
	Y6	Do you think participating in physical activity is unnecessary?	0 = No, 1 = Yes
	Y7	Do you worry about getting injured during physical activity?	0 = No, 1 = Yes
Family level	Y8	Do you have children?	0 = No, 1 = Yes
	Y9	Do you lack time for physical activity because of household responsibility?	0 = No, 1 = Yes
	Y10	Do you lack time for physical activity because of work demands?	0 = No, 1 = Yes
Community level	Y11	Do you find it convenient to engage in daily physical activity?	0 = No, 1 = Yes
	Y12	Do you feel there are not enough suitable facilities or venues for physical activity in your area?	0 = No, 1 = Yes
	Y13	Do you lack access to organized groups or teams for physical activity?	0 = No, 1 = Yes
	Y14	Do financial constraints make it difficult for you to maintain regular physical activity?	0 = No, 1 = Yes

#### Covariates

The covariates included age, sex, residence, education, occupation, and marital status.

### Statistical analysis

Statistical analyses were performed using SPSS software (version 30.0; IBM Corp., Armonk, NY, USA). Categorical variables were presented as frequencies and percentages. Sampling weights were calculated based on the age and urban–rural distribution of the national population aged 20–59 years from the corresponding census years, and weighted analyses were conducted to enhance national representativeness [25, 26]. Differences between groups were examined using χ² tests. To assess the correlates of interruption in PA behavior, generalized linear models with a logit link function were applied, with interruption in PA behavior (1 = yes, 0 = no) as a binomial outcome. Fourteen independent variables at the individual, family, and community levels were included, and sex, age, educational level, occupation type, and urban–rural residence were adjusted for as covariates. To evaluate model stability, multicollinearity was assessed using variance inflation factors (VIF) derived from linear regression. All VIF values were < 2.5, indicating no serious multicollinearity [27]. Model results were reported as odds ratios (ORs) with 95% confidence intervals (95% CI). To formally evaluate whether the strength of associations between multilevel correlates and PA interruption changed between 2015 and 2020, we performed a pooled analysis using the combined dataset. Survey year and interaction terms (survey year × each individual correlate) were included in the models. The significance of the change in associations over time was determined by the p-interaction of these interaction terms. All statistical tests were two-sided, and *p* < 0.05 was considered statistically significant.

## Results

Table 3 presents a comparison of the rates of interruption in PA behavior between 2015 and 2020 across demographic characteristics. The overall interruption rate increased from 6.8% in 2015 to 16.4% in 2020 (χ²=2596.72, *p* < 0.001). Significant differences in interruption rates were observed across age, sex, residence, marital status, education, and occupation (all *p* < 0.001). Across all demographic subgroups, the interruption rates showed a clear upward trend from 2015 to 2020.

**Table 3:** Demographic characteristics of the rates of interruption in physical activity behavior in 2015 and 2020 [n (%)]

Groups	Stratification	2020	2015	χ^2^	*p*
Total		10,724 (16.4)	3731 (6.8)	2596.72	< 0.001
Age group (years)	20–29	2633 (18.5)	1049 (7.7)	2615.15	< 0.001
	30–39	2789 (16.9)	1086 (7.9)	706.84	< 0.001
	40–49	2677 (15.7)	893 (6.5)	537.76	< 0.001
	50–59	2475 (14.2)	703 (5.1)	637.29	< 0.001
Sex	Male	6460 (19.6)	1950 (7.1)	687.75	< 0.001
	Female	4290 (13.3)	1781 (6.5)	1954.05	< 0.001
Residences	Urban	6869 (16.6)	2361 (8.7)	747.23	< 0.001
	Rural	3646 (15.3)	1370 (4.9)	878.77	< 0.001
Marital status	Unmarried	1827 (19.3)	624 (7.5)	1561.12	< 0.001
	Married	9776 (18.1)	3007 (6.7)	512.52	< 0.001
	Divorced	210 (16.8)	81 (7.0)	2821.13	< 0.001
	Widowed	67 (13.8)	19 (3.2)	52.60	< 0.001
Educational attainment	Primary school and below	1358 (13.2)	305 (3.5)	40.74	< 0.001
	Junior high school	3209 (14.2)	1022 (5.0)	560.26	< 0.001
	High school	2290 (15.6)	991 (7.5)	1004.48	< 0.001
	Undergraduate	2959 (17.2)	1365 (11.2)	442.07	< 0.001
	Postgraduate degree or above	87 (19.8)	48 (12.2)	204.30	< 0.001
Occupation	Occupation 1	5261 (13.5)	1239 (6.1)	8.45	0.004
	Occupation 2	817 (13.1)	579 (4.5)	734.99	< 0.001
	Occupation 3	2080 (17.1)	1089 (7.8)	460.88	< 0.001
	Occupation 4	766 (17.5)	542 (10.1)	522.57	< 0.001
	Occupation 5	664 (19.2)	282 (11.5)	114.74	< 0.001

Note: Chi-square test was used to compare differences in interruption rates across various categories of fitness behaviors between 2015 and 2020. The statistic is χ^2^

**Fig. 2:**
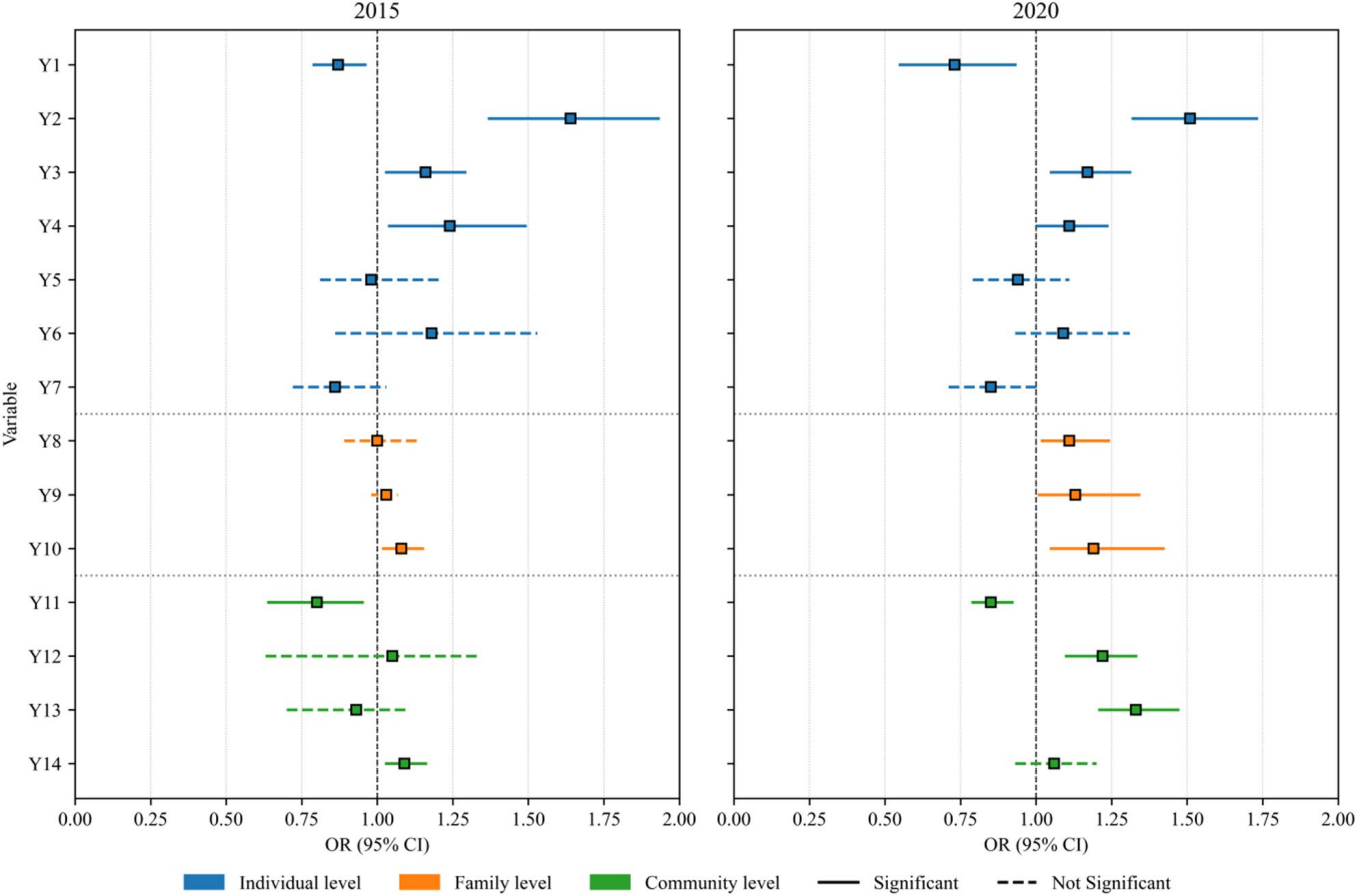
Correlates of interruption in physical activity behavior in 2015 and 2020. Note: Model was adjusted for age, sex, residence, education, occupation, and marital status

Further analyses were conducted using generalized linear models to examine the factors associated with interruption in PA behavior (Figure 2). The model demonstrated good fit (AIC = 74070.36, − 2LL = 36995.2), indicating stable estimates. After adjusting for covariates, several factors at the individual, family, and community levels were significantly associated with interruption in PA behavior. The results of the interaction items are shown in Table 4.

**Table 4:** Interaction effects between survey year and multilevel correlates of interruption in physical activity (2015 vs. 2020)

Variables	Interaction β	*p*-interaction	OR Ratio	95%CI
Y1	-0.786	< 0.001	0.456	0.414–0.502
Y2	0.559	< 0.001	1.749	1.429–2.140
Y7	0.999	< 0.001	2.715	2.089–3.528
Y8	0.438	< 0.001	1.549	1.397–1.718
Y9	0.319	< 0.001	1.376	1.239–1.527
Y11	-0.109	0.010	0.896	0.824–0.975
Y13	0.433	0.003	1.542	1.152–2.064

Note: OR Ratio represents the ratio of odds ratios between 2020 and 2015. The model was adjusted for age, sex, residence, education, occupation, and marital status. p-interaction < 0.05 indicates a statistically significant change in the association strength over time

At the individual level, lack of interest in PA and feeling lazy about PA engagement were positively associated with interruption. The odds ratio (OR) for lack of interest was 1.64 in 2015 (95% CI: 1.37–1.93, *p* < 0.001) and 1.51 in 2020 (95% CI: 1.32–1.73, *p* < 0.001). Lack of interest changed significantly between the two periods (OR ratio = 1.749, p-interaction < 0.001). Feeling lazy showed similar associations (2015 OR = 1.16, 95% CI: 1.03–1.29, *p* = 0.013; 2020 OR = 1.17, 95% CI: 1.05–1.31, *p* = 0.005). Perceiving a good physical condition was also associated with a higher likelihood of interruption in both surveys (2015 OR = 1.24, 95% CI: 1.04–1.49, *p* = 0.019; 2020 OR = 1.11, 95% CI: 1.00–1.24, *p* = 0.048). In contrast, receiving professional guidance was negatively associated with interruption (2015 OR = 0.87, 95% CI: 0.79–0.96, *p* = 0.002; 2020 OR = 0.73, 95% CI: 0.55–0.93, *p* < 0.001), with the protective effect strengthening significantly in 2020 (OR ratio = 0.456, p-interaction < 0.001).

At the family level, lack of time due to household responsibilities and lack of time due to work demands were associated with a higher likelihood of interruption in PA behavior. Notably, the interaction effects showed that the impact of household responsibility significantly increased in 2020 (OR ratio = 1.376, p-interaction < 0.001). Lack of time due to work demands was consistently and significantly associated with a higher likelihood of interruption in both survey years (2015 OR = 1.08, 95% CI: 1.02–1.15, *p* = 0.004; 2020 OR = 1.19, 95% CI: 1.05–1.42, *p* < 0.001). Childcare responsibility also showed a modest positive association in 2020 (OR = 1.11, 95% CI: 1.02–1.22, *p* = 0.016), representing a significant change in association compared to 2015 (OR ratio = 1.549, p-interaction < 0.001).

At the community level, perceived lack of convenient access was negatively associated with interruption (2015 OR = 0.80, 95% CI: 0.64–0.95, *p* = 0.014; 2020 OR = 0.85, 95% CI: 0.79–0.92, *p* < 0.001), with a statistically significant change in the association observed over time (OR ratio = 0.896, p-interaction = 0.010). In contrast, lack of suitable facilities (2020 OR = 1.22, 95% CI: 1.10–1.33, *p* < 0.001) and lack of organized groups or teams for interruption in PA behavior (2020 OR = 1.33, 95% CI: 1.21–1.47, *p* < 0.001) were positively associated with interruption, with the latter showing a significantly stronger association in 2020 compared to 2015 (OR ratio = 1.542, p-interaction = 0.003). Financial constraints were associated with interruption in 2015 (OR = 1.09, 95% CI: 1.03–1.16, *p* = 0.047) but were not statistically significant in 2020.

## Discussion

Using nationally representative data between 2015 and 2020, this study observed a notable increase in interruption in PA behavior among Chinese adults, alongside differentiated patterns across individual, family, and community factors. Because the two survey waves used consistent sampling and identical measurements, these differences likely reflect substantive social and behavioral changes rather than methodological variations. These findings reflect potential shifts in adults’ PA behaviors and suggest that internal motivational factors are becoming more relevant than in earlier periods.

### Increase in interruption in PA behavior

The national interruption rate increased by 9.6% between 2015 and 2020. A key contextual factor is the COVID-19 pandemic. Although the 2020 survey was conducted after strict restrictions had been lifted, earlier closures of sports venues, limitations on social gatherings, remote working arrangements, and disruptions to daily routines likely produced a substantial environmental shock that interrupted previously established PA habits [28, 29]. Such disruptions may have had lingering effects on adults’ ability to maintain regular PA, even when formal restrictions were no longer in place. In addition to pandemic-related shocks, long-term structural changes in daily life may have contributed to increasing vulnerability to interruption [30]. Adults in China experienced accelerating work rhythms and intensifying competitive pressures, which have been associated with growing constraints on time and energy allocation [31, 32]. The combined demands of work and family responsibilities may contribute to difficulties in PA behavioral continuity, which is consistent with earlier evidence linking long working hours and occupational fatigue to reduced PA levels. Although the ongoing implementation of national fitness policies has boosted the coverage of sports facilities [33], improvements in environmental resources alone may not correspond to proportional improvements in PA behavioral continuity. Built environment enhancements can support PA engagement [34]; however, strengthening individual motivation may not occur concurrently. This pattern aligns with a previous study emphasizing that intrinsic motivation and cognitive engagement remain essential components of PA behavioral continuity [35]. However, as this study was based on cross-sectional data, causal pathways could not be established. Therefore, the observed increase in interruptions should be interpreted as a likely combined influence of pandemic-related disruptions and broader lifestyle changes, rather than as definitive evidence of either factor alone. This interpretation reflects the temporal context of the 2020 wave, while maintaining appropriate caution regarding causal inference.

The increase in interruptions was particularly evident among adults aged 20–29 years, urban residents, and highly educated groups. Among younger adults, higher interruption rates may be related to competing demands of study and work, fast-paced lifestyles, and fragmented exercise times, as described in previous analyses of PA transitions in China [36]. Higher interruption rates among urban residents may reflect occupational competition, longer commuting times, and more sedentary work patterns [37]. Although individuals with higher educational levels generally exhibit stronger health awareness, heavy workloads and elevated mental stress may limit their ability to maintain regular PA [38]. Overall, the observed pattern suggests that improvements in the external environment do not necessarily translate into sustained behavioral engagement. These findings highlight the importance of paying greater attention to maintaining PA behavioral continuity among young adults and urban commuters for future health promotion.

### Analysis of factors associated with interruption in PA behavior

#### Individual-level correlates

At the individual level, Our interaction analysis further confirmed that the protective role of professional guidance became significantly more pronounced in 2020, suggesting that access to structured guidance and scientific exercise information is increasingly linked to greater confidence and planning in PA participation, as supported by prior evidence [39]. Lack of interest demonstrated a consistent positive association, indicating that insufficient intrinsic motivation remains an important barrier to maintaining PA behavioral continuity. According to self-determination theory, interest and enjoyment are central to sustaining long-term behaviors, and individuals with low affective involvement may be more likely to discontinue activity when experiencing stress or fatigue [40, 41]. Feeling lazy about PA engagement had a weaker association with interruptions in 2020. This pattern may align with increases in an exercise-friendly social atmosphere, expanded access to online exercise platforms, and the growth of home-based exercise options, which have lowered participation thresholds and may be particularly relevant for individuals with lower initial motivation [42]. Perceiving good health was associated with higher interruption rates in both survey years, suggesting that some adults underestimate the importance of continuous PA behavior and interpret their current health status as sufficient, consistent with earlier findings [43]. Most importantly, the negative association between professional guidance and interruption in PA behavior suggests that access to structured support is vital for behavioral maintenance. In China, such guidance primarily involves instruction from social sports instructors and the application of scientific exercise prescriptions [44]. Future public health strategies should ensure these resources are tailored to different educational backgrounds by providing simplified community-based instruction for individuals with lower educational attainment and data-driven feedback for those with higher education [45].

In contrast, worry about being ridiculed by others, perceiving PA as unnecessary, and worry about getting injured were not significantly associated with interruptions in either year. This pattern reflects the growing public acceptance of exercise and improvements in health communication, which may reduce social evaluation concerns, as well as improved facility safety and guidance systems that may alleviate injury-related concerns. While some psychological factors (feeling lazy) showed relatively small odds ratios, their significance is amplified when extrapolated to China’s vast population, where even a modest increase in interruption risk can affect millions. These findings underscore the importance of addressing even minor motivational lapses to ensure sustained national PA engagement. Overall, with continued improvements in social and environmental contexts, the main barriers to PA behavioral continuity among adults appear to have shifted from external constraints to internal psychological and motivational factors.

#### Family-level correlates

Family-level factors were also related to interruption in PA behavior. Childcare responsibility was positively associated with interruptions in 2020, which may correspond to evolving family structures and increased childcare responsibilities. As dual-earner households become more common and parenting demands intensify [46], PA may become deprioritized relative to work and childcare tasks. Notably, the association between lack of time due to household responsibilities and interruption in PA significantly intensified over the five-year period, suggesting that the unequal distribution of labor continues to limit opportunities for PA [47]. Although service convenience has reduced some physical burdens, disparities in household workload appear to remain, constraining exercise time [48]. Although the point estimates showed slight variations, the association with lack of time due to work demands remained a persistent and stable barrier across both survey waves, as indicated by the non-significant interaction effect. Previous research has described prolonged sedentary time and limited discretionary hours among working adults, which may contribute to difficulties in sustaining PA behavioral continuity [49]. Notably, the magnitude of this association weakened slightly in 2020, which may be related to the expansion of home-based exercise options and digital PA platforms that can increase worker flexibility [50, 51].

Overall, family-level factors appeared more prominent in 2020, potentially reflecting faster-paced lifestyles and increasing role demands that may limit the amount of time for continued PA behavior.

#### Community-level correlates

At the community level, perceived lack of convenient access was negatively associated with interruption in PA behavior in 2015 and 2020, suggesting a stable relationship between accessible environments and sustained PA behavioral continuity, consistent with earlier studies [52]. The promotion of the “15-min fitness circle” in recent years has contributed to more accessible exercise opportunities, particularly in urban settings [53, 54]. Previous studies have suggested that individuals who maintain exercise habits despite perceiving environmental barriers may possess higher intrinsic motivation, which buffers against behavioral interruption [55]. The significant change in the association for lack of organized groups or teams underscores the growing importance of social connectivity, reflecting a potential increase in the importance of social connection and group-based activities for maintaining PA behavior. With faster lifestyle rhythms and increased online socialization, individuals may rely more on group motivation and organized activities to ensure continuity of PA behavior [56]. When social support or a sense of belonging is limited, PA behaviors may become more challenging to maintain, underscoring the relevance of community social interactions and organized opportunities [57]. The association between financial constraints and interruption in PA behavior was significant in 2015, but not in 2020. This change may align with the expanded availability of low-cost exercise options, including online exercise classes and digital PA platforms [50, 58], which have lowered financial constraints to PA participation.

Overall, shifts in community-level factors appear to reflect the combined influence of improved physical environments and strengthened social support networks. Continued enhancements to community exercise resources, group-based opportunities, and social connectedness may be valuable in supporting PA behavioral continuity among adults.

### Limitations

This study had several limitations. First, it used a cross-sectional design, which does not allow the determination of causal relationships. Second, the interruption measure was based on self-reported data and may have been affected by a recall bias. Although individuals who had never engaged in PA were excluded, the survey did not directly confirm whether respondents had an established activity habit before the six-month period, which may have limited the precision of the measure. Third, the 2020 wave was conducted after the initial COVID-19 outbreak, and earlier disruptions to daily routines and access to exercise facilities may have influenced the reported behaviors. These effects cannot be separated from the broader lifestyle trends. Despite these limitations, the large sample size and multistage sampling enhanced the representativeness and robustness of our findings, though the resulting high statistical power warrants a cautious interpretation of small effect sizes in terms of their practical significance.

## Conclusions

Between 2015 and 2020, interruption in PA behavior among Chinese adults increased substantially, and the patterns of the associated factors underwent significant structural shifts. Interaction analyses confirmed that while some correlates remained stable, factors such as professional guidance, lack of interest, and domestic responsibilities showed significantly intensified associations with behavioral interruption over time. These findings suggest that efforts to support PA behavioral continuity benefit from approaches that consider both motivational processes and environmental resources, with an emphasis on strengthening self-regulatory capacities and social support systems, focusing on internal motivation, personalized guidance, and support for work-family balance to ensure sustained physical activity engagement.

## Data Availability

The data used in this study are derived from 2020 China National Fitness Activity Status Survey. Access to these data is restricted and requires formal application to the data owner, the General Administration of Sport of China (https://www.sport.gov.cn/n315/n329/). Due to the sensitive nature of the data and the potential for participant identification, the authors are unable to directly share the dataset with other researchers. However, inquiries regarding the data or potential collaborative research projects involving overlapping data are welcomeand can be directed to Yibo Gao (gaoyibo1019@163.com).
